# Editorial: Abnormal visual processing

**DOI:** 10.3389/fnins.2026.1907564

**Published:** 2026-07-24

**Authors:** Longqian Liu, Alexandre Reynaud, Jiawei Zhou

**Affiliations:** 1 Department of Ophthalmology, West China Hospital of Sichuan University, Chengdu, China; 2 Department of Ophthalmology and Visual Sciences, McGill University, Montreal, QC, Canada; 3 Department of Ophthalmology and Visual Sciences, Research Institute of the McGill University Health Centre, Montreal, QC, Canada; 4 Ningbo Eye Hospital, Wenzhou Medical University, Ningbo, China

**Keywords:** abnormal vision, amblyopia, exotropia, mild traumatic brain injury, vision

Vision is one of our most important senses, and its importance is growing in modern society. However, many people have abnormal visual processing due to various clinical conditions. These include amblyopia, myopia, strabismus and visual dysfunction resulting from an acquired brain injury, as well as retinal diseases and multiple sclerosis. These conditions can affect visual processing in the brain in different ways, dramatically affecting the way patients navigate their environment and their quality of life. Studying abnormal visual processing can reveal the neural mechanisms underlying these eye diseases and help us to better understand how the visual system works and adapts as a whole.

This Research Topic primarily focuses on scientific issues related to the neural mechanisms of abnormal visual disorders, such as amblyopia and intermittent exotropia, as well as the consequences of enucleation and mild traumatic brain injury. The aim is to understand the neural mechanisms underlying these conditions and how the brain adapts to them. This Research Topic presents three translational articles and two reviews about deficits, consequences, and clinical factors associated with these conditions.

Skrypek et al. used diffusion-weighted imaging and behavioral and survey data to assess reading speed, visual deficits and cerebral white matter integrity in veterans with and without mild traumatic brain injury. They discovered an association between smooth pursuit catch-up saccades and the total number of white matter lesions identified in the cerebral cortex. Their findings highlight the need for further research to establish the exact association between mTBI and reduced reading speed.

Zhao et al. investigated clinical factors associated with impaired near stereoacuity in children and adolescents with intermittent exotropia. Their findings show that impaired near stereoacuity affects over half of pediatric patients with intermittent exotropia, and that it is independently linked to anisometropia, longer disease duration, and greater deviation angles. The authors conclude that a routine assessment of near stereoacuity and refractive status is recommended to guide early intervention and management in this patient group.

Moro et al. used structural magnetic resonance imaging to measure the total pulvinar and total thalamus volumes of people who had undergone early monocular enucleation during postnatal maturation. They demonstrated that people with one eye had typical pulvinar and total thalamus volumes compared to binocular controls. The absence of pulvinar volume change in this subcortical region, which supports a variety of functions, contrasts with the changes observed in more sensory-specific areas. This provides evidence that neural plasticity is both regionally and functionally dependent.

This Research Topic then presents two reviews that aim to understand and guide the use of digital tools for assessing and treating visual conditions. Seraphim et al. conducted a literature review to determine the potential of automated analysis of brief visual electrophysiology signals in supporting medical interpretation in ophthalmology. They observed that artificial intelligence (AI) is being increasingly applied to such signals. AI can identify subtle or early dysfunction, harmonize interpretation across centers and support reproducible, clinician-independent pipelines for electrophysiology that are well-suited to high-volume clinics and large-scale screening. The authors emphasize the importance of selecting the most appropriate AI method for analysis. They conclude that the convergence of standardized acquisition protocols with advanced AI analysis could deliver more personalized, timely and objective assessments of visual system integrity in neuro-ophthalmic practice.

Finally, Zhou et al. present a comprehensive overview of the current state of research, key areas of focus, and emerging trends in amblyopia treatment, based on a bibliometric analysis of published literature. Their review reveals the current hotspots and future directions of amblyopia treatment research. Current research focuses on clinical trials of binocular treatment via various digital platforms for amblyopia patients. Their findings offer valuable insights for scientific and clinical research on amblyopia treatment.

This Research Topic aims to bring together cutting-edge research that sheds light on the neural mechanisms of abnormal visual processing. The focus is on the sensory experience of patients affected by visual conditions and the impact this has on their quality of life. The aim is to help design more accessible tools and identify novel therapeutic interventions that could mitigate or halt the progression of eye diseases.

